# Knowledge and Awareness of the Relationship Between Vitamin D Deficiency and Depression Among a Sample of Adults in Saudi Arabia

**DOI:** 10.7759/cureus.85555

**Published:** 2025-06-08

**Authors:** Ahmed Farid Al-Neklawy, Ghida Mohammed Aljohani, Muruj Abdullah Alrefaei, Alkhansaa Bajawi, Arwa Waleed Hammoda, Wed Khaled Tasji

**Affiliations:** 1 Department of Clinical Sciences, Fakeeh College for Medical Sciences, Jeddah, SAU; 2 Department of Anatomy and Embryology, Faculty of Medicine, Ain Shams University, Cairo, EGY; 3 College of Medicine, Fakeeh College for Medical Sciences, Jeddah, SAU

**Keywords:** deficiency, depression, knowledge, saudi arabia, vitamin d

## Abstract

Background: Vitamin D deficiency is a common nutritional issue that can lead to serious health consequences. Despite abundant sunlight, factors such as poor absorption, insufficient exposure, and increased demand contribute to this deficiency. Vitamin D plays a crucial role not only in bone health but also in mental well-being, with links to depression and other mood disorders.

Methods: This cross-sectional study involved a sample of 477 adults aged 18 years and above from Saudi Arabia. Participants were selected using simple random sampling. Data were collected through an online questionnaire. Statistical analysis, including descriptive and inferential methods, was conducted using IBM SPSS Statistics software, version 27 (IBM Corp., Armonk, NY).

Results: The results of the study indicate that among 477 participants surveyed, 323 (67.7%) reported having vitamin D deficiency, whereas 112 participants (23.5%) reported having experienced depression in the past. Of those who reported having experienced depression in the past, 80 persons (71.4%) felt that their depression was caused by vitamin D deficiency. The results also reveal that there is a gender difference in regards to vitamin D deficiency, with more females (230, 71.2%) being affected as compared to the male gender (93, 28.8%) (P=0.001). Further, results reveal that more Saudi (287, 88.9%) nationals were affected by vitamin deficiency as compared to 36 (11.1%) non-Saudis (p=0.015).

Conclusion: This study found that both vitamin D deficiency and depression are common among the population in Saudi Arabia. Many participants believed their experience of depression was linked to vitamin D deficiency, although this was based on self-reported perceptions rather than clinical diagnoses. While general awareness of vitamin D sources and benefits was relatively high, understanding of its broader health impacts was relatively low. The study also identified gender and nationality as important factors associated with vitamin D deficiency. Health professionals should incorporate structured patient education initiatives, such as dedicating an additional five to 10 minutes during clinical appointments to discuss vitamin D’s role in mental and physical health and developing informational brochures to be distributed in clinics and pharmacies.

## Introduction

Vitamin D is an essential fat-soluble vitamin that is necessary for several organs and systems, including bones and teeth, the immune system, brain health, and regulation of inflammation [1]. Although certain plant and animal foods provide some vitamin D, the most essential source of vitamin D is the sun [2]. Vitamin D is a crucial nutrient vital for overall health, including mental well-being. However, its deficiency is associated with various health complications, including mental disorders. Despite its significance, vitamin D deficiency remains widespread, both globally and in Saudi Arabia [3]. Vitamin D deficiency is attributable to such factors as poor absorption, insufficient exposure to vitamin D, aging, obesity, and increased demand. Additionally, some health conditions can also lead to vitamin D deficiency. For example, some gastrointestinal problems inhibit the absorption of vitamin D, leading to deficiency [3]. Research conducted to evaluate the prevalence of vitamin D deficiency in Saudi Arabia revealed that 63.5% of the population experienced this deficiency, while only 17.83% had normal vitamin D levels (≥30 ng/mL or 75 nmol/l) [4]. Despite the abundant sunshine in the region, vitamin D deficiency remains a prevalent nutritional concern in Saudi Arabia [4].

Numerous studies have highlighted that vitamin D holds greater significance as a nutrient than previously believed, particularly in its significant role in mental health [5-7]. A systematic review by Guzek et al. found that vitamin D plays a crucial role in impacting symptoms of depression, negative emotions, and overall quality of life [8]. Additionally, other studies have suggested that vitamin D affects several other brain functions, such as immunomodulation, neuroplasticity, and brain development [9, 10]. However, limited research has been undertaken to gauge the level of knowledge and awareness among the Saudi Arabian populace regarding the correlation between vitamin D deficiency and mental health challenges. Particularly, depression is a major challenge in Saudi Arabia, and strong links have been established between vitamin deficiency and depression [11]. This study aims to evaluate the knowledge and awareness about the relationship between vitamin D deficiency and depression among the Saudi Arabian population.

## Materials and methods

Study design and settings

This was a cross-sectional research design. The study primarily focused on assessing the knowledge and awareness levels regarding the relationship between vitamin D deficiency and depression among a sample of adults in Saudi Arabia.

Study subjects

Inclusion and Exclusion Criteria

The study focused on a sample of adults from the general population of Saudi Arabia. The inclusion criteria were adult population (aged above 18 years) of both genders (male and female) who consented to be part of the study. The exclusion criteria were minors (people below the age of 18 years) and those who were currently undergoing treatment for vitamin D deficiency or depression, or had a medical condition, or were taking medication known to affect vitamin D levels or mental health. For example, certain health conditions, such as Crohn’s disease and chronic kidney disease, can impair vitamin D absorption. Furthermore, medications like anticonvulsants (e.g., phenytoin) and glucocorticoids can interfere with vitamin D metabolism and contribute to deficiency.

Sample size

The sample size for this study was evaluated using the Cochrane sample size calculator. A 95% confidence interval (CI) was assumed, with an equal proportion of 0.5 and a margin of error of 5%. The minimum acceptable sample size was 384. However, our study involved 477 respondents to account for the non-response rate and incomplete questionnaires.

Sampling technique

The simple random sampling method was used to select the participants. The choice of this sampling technique was to ensure that each respondent had an equal chance of selection.

Data collection methods

Prior to the data collection exercises, a pilot study was conducted, and the data were analyzed to check for validity and reliability. The pilot study involved a smaller subset of participants, typically drawn from a similar demographic to the intended study population. This subset completed the survey or questionnaire, allowing researchers to assess the clarity of the questions and the feasibility and reliability of the questionnaire. The questionnaire was then administered online to the target population through social media like Telegram (Telegram FZ-LLC, Dubai, United Arab Emirates (UAE)), WhatsApp (Meta Platforms, Inc., Menlo Park, CA), and X (X Corp., Bastrop, TX). The questionnaire was composed of three main sections, namely, the social demographic section, a section containing knowledge about vitamin D, and a third section that had the awareness items towards vitamin deficiency and its effect on depression (Appendices A-D).

Data analysis plan

Data analysis involved both descriptive and inferential statistical analysis. Descriptive statistics were used to summarize the demographic characteristics of the study population, including age, gender, education level, marital status, etc. Inferential statistics, like multivariate regression analysis which were performed to assess the impact of vitamin D deficiency on multiple aspects. The continuous variables, like age, were presented using mean and standard deviation, while the categorical variables were presented in terms of numbers and frequencies. All the statistical analysis was carried out using IBM SPSS Statistics software, version 27 (IBM Corp., Armonk, NY), and the results were considered statistically significant at p < 0.05.

Ethical considerations

Ethical approval was obtained from the Research Ethics Committee at Fakeeh College of Medicine Sciences, Jeddah, Saudi Arabia (No: 144/IRB/2020). Some of the important ethical considerations included the respect of the patient's welfare, confidentiality, and informed consent. Respondents' rights to privacy and confidentiality were upheld throughout the study, with data being anonymized and securely stored to prevent any unauthorized access. Informed consent was obtained from participants or their legally authorized representatives before accessing their medical records for research purposes. Any potential risks or benefits associated with the study were clearly communicated to participants during the consent process. Lastly, researchers adhered to all applicable regulations and guidelines to safeguard the welfare and rights of the study participants while conducting this investigation.

## Results

Table 1 reveals that out of the 477 survey respondents, 306 (64.2%) were men and 171 (35.8%) were women. The average age of the participants was 35.30 years, with a 15.93 standard deviation (SD). A total of 404 participants, or 84.7%, were Saudi nationals; the remaining 73 participants, or 15.3%, were not from Saudi Arabia. In terms of education, 284 (59.5%) had attended college, 69 (14.5%) had only completed elementary school, 70 (14.7%) had earned a high school diploma, and 54 (11.3%) had earned a postgraduate degree. There was variation in the individuals' marital status: 216 (45.3%) were single, 239 (50.1%) were married, and 22 (4.6%) were divorced. Regarding family income, 66 people (13.8%) indicated they earned less than 5,000 Saudi Riyal (SAR), 135 people (28.3%) earned between 5,000 and 10,000 SAR, and 276 people (57.9%) earned more than 10,000 SAR. 

**Table 1:** Sociodemographic information of the participants (n=477) n: frequency; SD: standard deviation; SAR: Saudi Riyal

Variable	Category	n (%)
Gender	Male	171 (35.8%)
	Female	306 (64.2%)
Age	Mean ±SD	35.30±15.93
Nationality	Saudi	404 (84.7%)
	Non-Saudi	73 (15.3%)
Education	Elementary	69 (14.5%)
	High school	70 (14.7%)
	University	284 (59.5%)
	Post-graduate	54 (11.3%)
Marital Status	Single	216 (45.3%)
	Married	239 (50.1%)
	Divorced	22 (4.6%)
Family income level	Less than 5,000 SAR	66 (13.8%)
	5,000-10,000 SAR	135 (28.3%)
	More than 10,000 SAR	276 (57.9%)

According to Table 2, 154 (32.3%) of the respondents had never had a vitamin D shortage, whereas 323 (67.7%) of the participants had. Regarding depression, 365 individuals (76.5%) claimed never to have experienced depression, whereas 112 participants (23.5%) reported having experienced depression in the past. Of those who reported having experienced depression in the past, 80 participants indicated that they personally believed their depression was due to vitamin D deficiency. This was a self-reported perception based on individual experience, not a clinical diagnosis. 

**Table 2:** Prevalence of vitamin D deficiency and depression among the participants (n=477) Data have been presented as frequency and percentage.

Variable	Category	n (%)
Have you had vitamin D deficiency before?	Yes	323 (67.7%)
	No	154 (32.3%)
Have you had depression before?	Yes	112 (23.5%)
	No	365 (76.5%)
Have you had depression due to vitamin D deficiency before? (If your answer was yes to the previous question)	Yes	80 (71.4%)
	No	32 (28.6%)

Table 3 reveals that 247 participants (51.8%) evaluated their knowledge positively. A total of 419 participants, or 87.8%, knew that exposure to sunlight promotes the skin's synthesis of vitamin D. Furthermore, 319 (66.9%) respondents agreed that living in cloudy regions increases the risk of vitamin D insufficiency. A number of the participants, 340 (71.3%), acknowledged that using sunscreen creams can exacerbate vitamin D deficiency. Moreover, 324 people (67.9%) thought that a diet low in fat could result in a vitamin D deficit. In terms of skin tone, 326 participants (68.3%) believed that darker skin types are more vulnerable to vitamin D deficiency than lighter skin types. Furthermore, 321 (67.3%) respondents indicated that compared to non-vegetarians, vegetarians are more likely to be vitamin D deficient. Regarding absorption, 322 people (67.5%) indicated that inadequate vitamin D levels might be connected to issues with absorption. Just 153 (32.1%) people thought there was a link between vitamin D deficiency and aging. In regard to obesity, 272 (57.0%) of the participants believed that low vitamin D levels and obesity were related. 

**Table 3:** Knowledge and awareness regarding vitamin D deficiency among the participants (n=477) Data have been presented as frequency and percentage.

Variable	Category	n(%)
How will you evaluate your knowledge about vitamin D deficiency?	Excellent	97 (20.4%)
	Good	150 (31.4%)
	Poor	230 (48.2%)
Sun exposure encourages production of vitamin D in the skin	Yes	419 (87.8%)
	No/I don’t know	58 (12.2%)
People residing in cloudy areas are more prone to vitamin D deficiency	Yes	319 (66.9%)
	No/I don’t know	158(33.1%)
Use of sunscreen creams may be a cause of vitamin D deficiency	Yes	340 (71.3%)
	No/I don’t know	137(28.7%)
A fat-free diet may be a cause of vitamin D deficiency	Yes	324 (67.9%)
	No/I don’t know	153 (32.1%)
Dark skin is more prone to vitamin D deficiency than fairer skin	Yes	326 (68.3%)
	No/I don’t know	151 (31.7%)
Vegetarians are more likely to have a vitamin D deficiency than non-vegetarians	Yes	321 (67.3%)
	No/I don’t know	156 (32.7%)
Do you think vitamin D deficiency is related to poor absorption?	Yes	322 (67.5%)
	No/I don’t know	155 (32.5%)
Is there a connection between vitamin D deficiency and aging?	Yes	153 (32.1%)
	No/I don’t know	324 (67.9%)
Is there a connection between vitamin D deficiency and obesity?	Yes	272 (57.0%)
	No/I don’t know	205 (43.0%)

Table 4 shows that 265 (55.6%), 148 (31.0%), and 64 (13.4%) of the participants rated their comprehension of vitamin D deficiency-related depression symptoms and suitable management as excellent, good, or poor, respectively. While 311 individuals (65.2%) believed that sadness can result in trouble falling or staying asleep or excessive sleeping, 282 participants (59.1%) claimed that depression linked to vitamin D deficiency might induce poor appetite or overeating. Furthermore, 388 (81.3%) individuals thought that depression associated with a vitamin D deficiency could result in fatigue or low energy, and 306 (64.2%) people thought that sadness could create emotions of hopelessness or depression. In terms of additional symptoms, 228 participants (47.8%) stated that depression associated with vitamin D deficiency can result in feeling bad, failure, or having let oneself or one's family down; 361 people (75.7%) thought it can produce restlessness, anxiety, or fidgetiness. Of the participants, 149 (31.2%) thought that depression caused by a vitamin D deficiency could result in suicidal thoughts or cause death, 244 (51.2%) thought it could cause excessive weight loss or gain, and 272 (57.0%) thought it could induce headaches or back discomfort. The participants, as management strategies, brought up the following choices: 127 picked psychotherapy (26.6%), depressive medication (86, 18.0%), vitamin D supplementation was picked by 387 (81.1%), increased sun exposure by 302 (63.3%), and vitamin D-fortified food consumption by 370 (77.6%).

**Table 4:** Participants' knowledge and awareness regarding symptoms and proper management of depression related to vitamin D deficiency (n=477) Data have been presented as frequency and percentage.

Variable	Category	n(%)
How will you evaluate your knowledge about symptoms and proper management of depression related to vitamin D deficiency?	Excellent	64 (13.4%)
	Good	265 (55.6%)
	Poor	148 (31.0%)
Do you think depression related to vitamin D deficiency can cause poor appetite or overeating?	Yes	282 (59.1%)
	No/I don’t know	195 (40.9%)
Do you think depression related to vitamin D deficiency can cause trouble falling or staying asleep, or sleeping too much?	Yes	311 (65.2%)
	No/I don’t know	166 (34.8%)
Do you think depression related to vitamin D deficiency can lead to feelings of hopelessness or feeling down?	Yes	306 (64.2%)
	No/I don’t know	171 (35.8%)
Do you think depression related to vitamin D deficiency can lead to feeling tired or having little energy?	Yes	388 (81.3%)
	No/I don’t know	89 (18.7%)
Do you think depression related to vitamin D deficiency can cause feeling bad about oneself, or that you are a failure, or have let yourself or your family down?	Yes	228 (47.8%)
	No/I don’t know	249 (52.2%)
Do you think depression related to vitamin D deficiency can cause trouble concentrating on things such as reading or watching television?	Yes	270 (56.6%)
	No/I don’t know	207 (43.4%)
Do you think depression related to vitamin D deficiency can cause feelings of restlessness, uneasiness, or being fidgety?	Yes	361 (75.7%)
	No/I don’t know	116 (24.3%)
Do you think depression related to vitamin D deficiency can lead to thoughts of death or suicide?	Yes	149 (31.2%)
	No/I don’t know	328 (68.8%)
Do you think depression related to vitamin D deficiency can cause excessive weight loss or weight gain?	Yes	244 (51.2%)
	No/I don’t know	233 (48.8%)
Do you think depression related to vitamin D deficiency can cause headaches or back pain?	Yes	272 (57.0%)
	No/I don’t know	205 (43.0%)
How do you think depression related to vitamin D deficiency can be managed? (You can choose more than 1 item)	Psychotherapy	127 (26.6%)
	Antidepressant medication	86 (18.0%)
	Taking vitamin D supplements	387 (81.1%)
	Increasing your sun exposure	302 (63.3%)
	Eating foods that contain vitamin D or are fortified with Vitamin D	370 (77.6%)

Table 5 shows that the results from multivariate regression analysis reveal that there is a gender difference in regards to vitamin deficiency, with more females (230, 71.2%) being affected as compared to the male gender (93, 28.8%) (P=0.001). Further, results reveal that more Saudi nationals (287, 88.9%) were affected by vitamin deficiency as compared to 36 (11.1%) non-Saudis (p=0.015). 

**Table 5:** Prevalence and multivariate regression analysis of vitamin D deficiency Data have been presented as n, % and, Mean ±SD; multivariate regression analysis was used; P<0.05* was statistically significant; SAR: Saudi Riyal

Category	Yes n (%)	No n (%)	Coefficients	t	P-value*
Gender			0.301	6.181	0.001
Male	93 (28.8%)	78 (50.6%)			
Female	230 (71.2%)	76 (49.4%)			
Age			0.003	1.175	0.241
Mean ±SD	35.30±15.93	35.30±15.93			
Nationality			-0.157	-2.449	0.015
Saudi	287 (88.9%)	117 (76.0%)			
Non-Saudi	36 (11.1%)	37 (24.0%)			
Education			0.035	1.288	0.198
Elementary	39 (12.1%)	30 (19.5%)			
High school	47 (14.6%)	23 (14.9%)			
University	202 (62.5%)	82 (53.2%)			
Post-graduate	35 (10.8%)	19 (12.3%)			
Marital status			0.066	1.176	0.240
Single	133 (41.2%)	83 (59.9%)			
Married	171 (52.9%)	68 (44.2%)			
Divorced	19 (5.9%)	3 (1.9%)			
Income			0.032	0.984	0.326
Less than 5,000 SAR	37 (11.5%)	29 (18.8%)			
5,000-10,000 SAR	94 (29.1%)	41 (26.6%)			
More than 10,000 SAR	194 (59.4%)	84 (54.5%)			

## Discussion

The main objective of this study was to investigate the knowledge and awareness regarding the relationship between vitamin D deficiency and depression among the population of Saudi Arabia. The findings of the study reveal that among the participants surveyed, 323 (67.7%) reported having vitamin D deficiency, whereas 112 participants (23.5%) reported having experienced depression in the past. Of those who reported having experienced depression in the past, 80 persons (71.4%) explicitly reported having been depressed as a result of the vitamin D deficiency. Similar to this finding, a study by Babelghaith et al. found that most of the respondents surveyed indicated that they had a vitamin D deficiency [12]. Further, a study by AlGarni et al. found that most of the respondents surveyed revealed that they were experiencing depression related to vitamin D deficiency [13].

According to the findings of this study, most of the respondents, 311 (65.2%), believed that depression associated with vitamin D deficiency can result in challenges of falling or staying asleep or excessive sleeping. Moreover, 282 participants (59.1%) claimed that depression linked to vitamin D deficiency might induce poor appetite or overeating. Furthermore, 388 (81.3%) individuals thought that depression associated with a vitamin D deficiency could result in fatigue or low energy, and 306 (64.2%) people thought that depression could create emotions of hopelessness. In terms of additional symptoms, 228 participants (47.8%) stated that depression associated with vitamin D deficiency could result in feeling bad, failure, or having let oneself or one's family down, while 361 respondents (75.7%) indicated that it could result in restlessness, anxiety, or fidgetiness. Additionally, 149 (31.2%) of the respondents indicated that depression caused by a vitamin D deficiency could result in suicidal thoughts or cause death; 244 (51.2%) thought it could cause excessive weight loss or gain; and finally, more than half, 272 (57.0%), thought it could induce headaches or back discomfort. This finding concurs with the results obtained in a study by Menon et al., which found that the depression associated with deficiency of vitamin D involved feeling bad, feeling a failure, or having the family down [14].

In regards to the sources of vitamin D, the findings of this study indicate that most of the participants, 387 (81.1%), were aware of vitamin D supplementation as a source of vitamin D, followed by vitamin D-fortified food consumption with 370 (77.6%) and then increased sun exposure by 302 (63.3%). On the other hand, a few 127 indicated psychotherapy (26.6%) and depressive medication 86 (18.0%) as the source of vitamin D. This is in line with a study by Hassan et al., which revealed the major source of vitamin D is the consumption of vitamin D supplementation and vitamin D-fortified food consumption [15]. A study by Gholamzad et al. also supports this finding in that their study found the consumption of food rich in vitamin D and the uptake of vitamin D supplements is the quickest way of recovering from vitamin D deficiency among the affected individuals [16].

The assessment of the prevalence of vitamin D deficiency among different demographic groups revealed that there is a gender difference in regard to vitamin deficiency, with more females (230, 71.2%) being affected as compared to males (93, 28.8%) (P=0.001). Further, the study finding reveals that more Saudi nationals (287, 88.9%) were affected by vitamin deficiency as compared to non-Saudis (36, 11.1%) (p=0.015). This concurs with the finding obtained in a systematic review conducted by Fitzgerald et al., which revealed that the female gender showed a significantly higher proportion of vitamin D deficiency compared to the male gender [17].

Finally, in terms of the attitudes and beliefs towards sunlight exposure and its role in vitamin D synthesis, the finding of this study revealed that 419 participants, or 87.8%, knew that exposure to sunlight promotes the skin's synthesis of vitamin D. Furthermore, 319 (66.9%) respondents agreed that living in cloudy regions increases the risk of vitamin D deficiency. Additionally, a number of the participants, 340 (71.3%), acknowledged that using sunscreen creams can exacerbate vitamin D deficiency. In terms of skin color, 326 participants (68.3%) believed that darker skin color is more vulnerable to vitamin D deficiency than lighter skin color. These findings are aligned with the findings in a study by Subedee et al., which found that exposure to sunlight promotes the skin's synthesis of vitamin D [18]. Similarly, a study by Kagotho et al. found that living in cloudy regions increases the risk of vitamin D deficiency [19]. This implies that the majority of the participants had good knowledge of vitamin D deficiency and the factors contributing to it.

In terms of these study limitations, not much information was obtained because closed-ended questionnaires were used to collect data. Further, the use of online data collection methods subjected the study to selection bias. Therefore, comprehensive research addressing the limitations of this study is required to generalize the findings to a broader context.

## Conclusions

The knowledge and awareness of vitamin D varied among the respondents surveyed. However, a number of the participants were knowledgeable about vitamin D deficiency and its implications. Further, this study has noted that gender and nationality play significant roles in determining vitamin D deficiency. Therefore, health professionals should stress to patients and their families the value of vitamin D and the effects of vitamin D deficiency. They can do this by scheduling extra time in the clinic to educate patients and their families or by organizing frequent community awareness campaigns.

## Appendices

Appendix A 

**Table 6:** Sociodemographic information of the participants

Question	Options
Gender	Male
	Female
Age	18-29 years
	30-49 years
	50-64 years
	65 or older
Nationality	Saudi
	Non-Saudi
Region of residence	Eastern region
	Western region
	Central region
	Southern region
	Northern region
Education level	Elementary
	Intermediate school
	High school
	University
	Post-graduate
Marital status	Single
	Married
	Widowed
	Divorced

Appendix B 

**Table 7:** Prevalence of vitamin D deficiency and depression among the participants

Variable	Category
Have you had vitamin D deficiency before?	Yes
	No
Have you had depression before?	Yes
	No
Have you had depression due to vitamin D deficiency before? (If your answer was yes to the previous question)	Yes
	No

Appendix C 

**Table 8:** Knowledge and awareness about vitamin D deficiency among the participants

Variable	Category
How will you evaluate your knowledge about vitamin D deficiency?	Excellent
	Good
	Poor
Sun exposure encourages production of vitamin D in the skin	Yes (Correct)
	No/I don’t know
People residing in cloudy areas are more prone to vitamin D deficiency	Yes (Correct)
	No/I don’t know
Use of sunscreen creams may be a cause of vitamin D deficiency	Yes (Correct)
	No/I don’t know
A fat-free diet may be a cause of vitamin D deficiency	Yes (Correct)
	No/I don’t know
Dark skin is more prone to vitamin D deficiency than fairer skin	Yes (Correct)
	No
	I don’t know
Vegetarians are more likely to have vitamin D deficiency than nonvegetarians	Yes (Correct)
	No/I don’t know
Do you think vitamin D deficiency is related to poor absorption?	Yes (Correct)
	No/I don’t know
Is there connection between vitamin D deficiency and aging?	Yes (Correct)
	No/I don’t know
Is there connection between vitamin D deficiency and obesity?	Yes (Correct)
	No/I don’t know

Appendix D 

**Table 9:** Knowledge and awareness about symptoms and proper management of depression related to vitamin D deficiency among the participants

Variable	Category
How will you evaluate your knowledge about symptoms and proper management of depression related to vitamin D deficiency?	Excellent
	Good
	Poor
Do you think depression related to vitamin D deficiency can cause poor appetite or overeating?	Yes (Correct)
	No/I don’t know
Do you think depression related to vitamin D deficiency can cause trouble falling or staying asleep, or sleeping too much?	Yes (Correct)
	No/I don’t know
Do you think depression related to vitamin D deficiency can lead to hopelessness of feeling down?	Yes (Correct)
	No/I don’t know
Do you think depression related to vitamin D deficiency can lead to feeling tired or having little energy?	Yes (Correct)
	No/I don’t know
Do you think depression related to vitamin D deficiency can cause feeling bad about oneself or that you are a failure or have let yourself or your family down?	Yes (Correct)
	No/I don’t know
Do you think depression related to vitamin D deficiency can cause trouble concentrating on thing such as reading or watching television?	Yes (Correct)
	No/I don’t know
Do you think depression related to vitamin D deficiency can cause feeling restlessness, uneasy or fidgety?	Yes (Correct)
	No/I don’t know
Do you think depression related to vitamin D deficiency can lead to thoughts of death or suicide?	Yes (Correct)
	No/I don’t know
Do you think depression related to vitamin D deficiency can cause excessive weight loss or weight gain?	Yes (Correct)
	No/I don’t know
Do you think depression related to vitamin D deficiency can cause headaches or back pains?	Yes (Correct)
	No/I don’t know
How do you think depression related to vitamin D deficiency can be managed? (You can choose more than 1 item)	Psychotherapy (Correct)
	Antidepressant medication (Correct)
	Taking vitamin D supplements (Correct)
	Increasing your sun exposure (Correct)
	Eating foods that contain vitamin D or are fortified with vitamin D (Correct)
